# New Vaccine Platforms—Novel Dimensions of Economic and Societal Value and Their Measurement

**DOI:** 10.3390/vaccines12030234

**Published:** 2024-02-24

**Authors:** Philip O. Buck, Dumingu Aparna Gomes, Ekkehard Beck, Noam Kirson, Matthew Mattera, Stuart Carroll, Bernhard Ultsch, Kavisha Jayasundara, Mathieu Uhart, Louis P. Garrison, Jr.

**Affiliations:** 1 Moderna Inc., Cambridge, MA 02139, USA; philip.buck@modernatx.com (P.O.B.); ekkehard.beck@modernatx.com (E.B.); 2 Analysis Group, Inc., Menlo Park, CA 94025, USA; aparna.gomes@analysisgroup.com; 3 Analysis Group, Inc., Boston, MA 02199, USA; noam.kirson@analysisgroup.com; 4 Analysis Group, Inc., New York City, NY 10036, USA; matthew.mattera@analysisgroup.com; 5 Moderna Inc., London SW1E 6DE, UK; stuart.carroll@modernatx.com; 6 Moderna Germany GmbH, 81671 München, Germany; bernhard.ultsch@modernatx.com; 7 Moderna Inc., Toronto, ON M5V 0C3, Canada; kavisha.jayasundara@modernatx.com; 8 Moderna Inc., 75007 Paris, France; mathieu.uhart@modernatx.com; 9 School of Pharmacy, University of Washington, Seattle, WA 98195, USA; lgarrisn@uw.edu; 10 Global Health Economics LLC, Mercer Island, WA 98040, USA

**Keywords:** vaccines, NITAGs, health technology assessment, value framework, value measurement, next-generation vaccine platforms

## Abstract

The COVID-19 pandemic’s dramatic impact has been a vivid reminder that vaccines—especially in the context of infectious respiratory viruses—provide enormous societal value, well beyond the healthcare system perspective which anchors most Health Technology Assessment (HTA) and National Immunization Technical Advisory Group (NITAG) evaluation frameworks. Furthermore, the development of modified ribonucleic acid-based (mRNA-based) and nanoparticle vaccine technologies has brought into focus several new value drivers previously absent from the discourse on vaccines as public health interventions such as increased vaccine adaptation capabilities, the improved ability to develop combination vaccines, and more efficient vaccine manufacturing and production processes. We review these novel value dimensions and discuss how they might be measured and incorporated within existing value frameworks using existing methods. To realize the full potential of next-generation vaccine platforms and ensure their widespread availability across populations and health systems, it is important that value frameworks utilized by HTAs and NITAGs properly reflect the full range of benefits for population health and well-being and cost efficiencies that these new vaccines platforms provide.

## 1. Introduction

It has been widely recognized that economic assessments of the value of health technologies (including diagnostics, therapeutic interventions, and preventive interventions) introduce a host of important elements that may not be captured in traditional cost-effectiveness analyses (CEAs) of therapeutic technologies. For this Special Issue on The Health Economic Value of Vaccines, we review three novel dimensions of value that the development of modified ribonucleic acid-based (mRNA-based) and nanoparticle vaccine technologies has brought into focus: increased vaccine adaptation capabilities, improved ability to develop combination vaccines, and more efficient vaccine manufacturing and production processes.

The proper clinical and economic evaluation of vaccination programs necessitates recognition of both the direct clinical value, as well as the broader societal benefits conferred beyond healthcare systems or sectors—such as health system resilience and a broad range of non-health macroeconomic benefits (such as of human capital accumulation and labor market productivity) [1,2,3,4]. These considerations were reflected in the Professional Society for Health Outcomes and Research (formerly International Society of Pharmacoeconomics and Outcomes Research, ISPOR) 2018 Task Force Report on best practices in the economic assessment of vaccination programs, as well as in the World Health Organization’s (WHO) recently published Full Value of Vaccine Assessments (FVVA) framework and the Asia-Pacific Economic Cooperation (APEC) Regional Dashboard on Vaccination Across the Life-Course [5,6,7]. Failing to include such elements in CEAs or formal Health Technology Assessments (HTAs) risks undervaluing vaccine technologies, ultimately reducing manufacturer incentives to invest in research and development (R&D) to develop vaccines for diseases with sustained unmet need [8]. 

The COVID-19 pandemic’s dramatic worldwide impact, of course, has been a vivid reminder that vaccines—especially in the context of infectious respiratory viruses—can provide huge societal value that goes well beyond the healthcare system perspective that anchors most HTAs. A large and growing body of research has repeatedly illustrated this over the last three years, pointing to the importance of expanded value frameworks and taking a broader context in the evaluation of vaccines, whether for public health policy recommendations of National Immunization Technical Advisory Groups (NITAGs) or market access and reimbursement decisions influenced by HTA agencies [1,9,10,11,12,13,14]. 

The pandemic has also highlighted the value of new vaccine development platforms that can generate novel economic value. The development of mRNA-based and nanoparticle vaccine technologies has brought into focus several new value drivers previously absent from the assessment of vaccines as public health interventions, which should be considered in addition to the well-documented direct clinical value from their proven efficacy [14,15,16,17]. Three such aspects include: (1) increased vaccine adaptation capabilities that can facilitate development of novel vaccines for pandemic viruses and later strain matching for seasonal respiratory viruses; (2) greater ability to combine vaccines against multiple diseases into a single injection, leading to increased administration efficiencies and reduced patient burden; and (3) more efficient vaccine manufacturing and production processes that lead to a more secure supply with lower vulnerability to global supply chain shifts. These three enhanced capabilities are arguably examples of what the ISPOR Value Flower would categorize as a type of insurance value called “health risk protection”: all health plan members—i.e., potential patients—are better off knowing new production approaches are available to address threats by any novel viruses.

These areas have received considerably less attention in the context of value assessment and have yet to be meaningfully quantified in CEAs by HTA bodies [11,12]. Below, we describe each of these unique value dimensions and discuss how they might be captured within existing or adapted value frameworks that inform HTA and NITAG decision making, and what challenges may lie ahead in incorporating them.

## 2. Increased Vaccine Adaptation Capabilities

The periodic emergence of new zoonotic viruses and variants has threatened to render existing vaccines less effective [18,19]. Most recently, the value of next-generation vaccine platforms’ increased vaccine adaptation capabilities was demonstrated with the development of vaccines against COVID-19. Fast, large-scale vaccine production in pandemic conditions was not feasible with classical vaccine platforms. As discussed by van Riel and de Witt (2020), growing large quantities of virus under biosafety level 3 (BSL3) conditions for a whole-inactivated virus, meeting extensive safety testing requirements to ensure live-attenuated viruses are safe and do not easily revert to wild type, and simultaneous production of several recombinant proteins for virus-like particle vaccines are not feasible with protein-based and virus-based vaccine platforms [18]. Next-generation vaccine platforms with increased adaptation capabilities are filling this unmet need. The main advantage of next-generation vaccine platforms is that vaccines can be developed using viral protein(s) coding sequence information alone (instead of needing to have the ability to culture the virus with classical vaccine platforms), which makes these next-generation platforms highly adaptable and speeds up vaccine development considerably [20,21]. For instance, only 63 days passed from the publication of the SARS-CoV-2 genomes on 10 January 2020 to the United States National Institutes of Health (NIH) announcement that the first human participant in its Phase 1 study for mRNA-1273 was dosed [22]. 

The increased vaccine adaptation capabilities of next-generation vaccine platforms (such as nanoparticle and mRNA- based vaccine technologies) have also allowed researchers to continue meeting the evolving profile of COVID-19. On 15 June 2023, the United States Food and Drug Administration’s (FDA) Vaccines and Related Biological Products Advisory Committee (VRBPAC) recommended SARS-CoV-2 strain(s) for updated COVID-19 vaccines for use in the United States, beginning in the fall of 2023 [23]. On 11 September 2023, the FDA approved and authorized for emergency use updated COVID-19 vaccines, formulated to more closely target currently circulating variants [24]. In fact, only vaccines produced using next-generation platforms are currently approved to combat new variants of COVID-19, and the United States Department of Health and Human Services (DHHS) $500 million investment in Project NextGen’s first round of next-generation vaccine candidates (building on the $1.4 B awarded in August 2023) indicates the immense potential next-generation vaccine platforms hold [16,25,26]. 

Vaccine development for other circulating respiratory viruses, such as seasonal influenza, also require adaptation to novel and emerging strains [27,28]. Traditional vaccine development processes have required researchers to recommend the viruses for quadrivalent vaccines to the WHO approximately 6 months prior to ‘flu season’ [29,30,31]. Because viruses (including influenza viruses) continuously adapt and evolve, the WHO uses global surveillance data from the previous 5 to 8 months to update their vaccine recommendations each February for the Northern Hemisphere and each September for the Southern Hemisphere—approximately 6 to 9 months before vaccine deployment [28,30]. The lengthy lead time is also associated with traditional vaccine development approaches needing live cell cultures to grow large amounts of the virus that are then inactivated or attenuated [20,30,31,32,33]. The implication of this prolonged development timeline is that predictions for the upcoming season’s strains (which are based on analyses of region-specific epidemiologic, genetic, and antigenic data, as well as vaccine effectiveness models) are at risk of becoming outdated [30,34]. 

Vaccine mismatches resulting in reduced vaccine effectiveness have occurred in recent years, when circulating influenza strains changed after decisions were made about vaccine composition [30]. For example, vaccine effectiveness was only 13% against influenza A (H3N2) during the 2014–2015 influenza season in the United States. This was because over 80% of the identified influenza A (H3N2) viruses differed from those included in the vaccine [30,35]. Even in seasons where influenza vaccines are considered to be close matches to the circulating influenza viruses, vaccine effectiveness only ranges from 40 to 60% [30]. In the 2016–2017 Northern Hemisphere influenza vaccine, adjustments were made to incorporate a new influenza A (H3N2) component. Despite this adjustment and the similarity between the vaccine strain and the majority circulating virus strains, the initial effectiveness estimate was 42% overall and merely 34% against influenza A (H3N2) viruses, as reported by the CDC [30,36]. In contrast, the faster adaptation capabilities of mRNA-based vaccines allow researchers to gather more data proximate to ‘flu season’ as the basis for their recommendations [20,37,38,39]. However, to leverage next-generation platforms to develop vaccines that better match circulating influenza virus strains, region-specific health authorities and NITAGs need to issue a strain recommendation later in the year than the WHO recommendation—similar to how the FDA issued a COVID-19 strain recommendation for the United States in June 2023 after the WHO issued their recommendations in February 2023 [23,24]. 

In terms of value assessment, NITAGs and HTAs already take into consideration a vaccine’s anticipated efficacy in their decision-making processes [40,41,42,43,44,45,46,47,48]. For seasonal vaccines, the increased clinical efficacy associated with later strain-matching capabilities of mRNA vaccine platforms can be estimated using existing evaluation processes and incorporated into the periodic CEA and value assessments conducted by HTAs and NITAGs. While the incremental efficacy of mRNA or nanoparticle vaccines over egg-based vaccines may not be known with certainty, sensitivity and scenario analyses can be used to test a range of clinically driven assumptions.

## 3. Improved Ability to Develop Combination Vaccines

Combining multiple vaccines into a single formulation has long been a public health goal in the development of vaccines against infectious diseases [49,50]. Advantages of combination vaccines include a reduction in the number of injections that a care provider has to administer and a patient has to receive, simpler vaccine schedules, decreased vial usage and biologic waste, and lower distribution and storage costs (which is particularly important when reaching particularly vulnerable or remote populations) [49,51,52,53]. These conveniences, in turn, lead to greater patient compliance, higher vaccination rates, improved vaccine coverage, lower administration costs, and lower overall hospitalization and physician services costs to the health system and patient population [50,53,54,55,56,57]. For instance, in a study using 2012 National Immunization Survey (NIS) data, vaccination completion was evaluated at ages 8, 18, and 24 months, with compliance assessed specifically at 24 months. Results indicated that children who received at least one combination vaccine (86%) demonstrated higher rates of completion (i.e., 69% patients received all doses of a vaccine) and compliance with the full vaccine series at 24 months (i.e., 24% received each dose of the vaccine series during the recommended age window) compared to those who received only single-antigen vaccines (50% completion rate and 13% compliance with full vaccine series during the recommended age window). Furthermore, the receipt of combination vaccines was associated with increased odds of completing all recommended vaccinations at 24 months (odds ratio: 2.5; *p* < 0.001) and receiving all vaccinations at age-appropriate times (odds ratio: 2.2; *p* < 0.001) [54]. 

However, concerns about (a) combining multiple viruses or bacteria into a single vaccine, (b) the potential for interference between vaccine components, and (c) ensuring a measured immune response against multiple antigens have in the past made developing combination vaccines challenging [49,58,59,60]. mRNA-based combination vaccines pose less risks and concerns as scientists can encode multiple antigens into a single mRNA sequence to target multiple viruses or multiple strains of the same virus [60,61,62]. In fact, several combination vaccine candidates against seasonal respiratory diseases utilizing next-generation vaccine platforms (e.g., Flu-COVID, Flu-RSV, and Flu-COVID-RSV) are currently being tested in Phase 2/3 clinical programs and vaccines with multiple strains of the same virus are already available to the public (e.g., FluMist Quadrivalent) [60,63]. mRNA-based vaccine development has even opened the doors to targeting diseases caused by latent viruses (e.g., cytomegalovirus [CMV] and human immunodeficiency virus [HIV]), enteric viruses (e.g., noroviruses), and bacteria (e.g., Lyme disease) [64,65,66,67,68,69]. 

Furthermore, combination vaccines are likely to enhance vaccination rates across multiple diseases, resulting in population-wide benefits such as greater herd protection and lower burden across multiple diseases [50,54,55,70,71]. Direct benefits arise from improved health outcomes for vaccinated individuals and cost-savings from reduced visits and streamlined distribution efforts (e.g., transportation costs avoided) [53,55]. Indirect benefits include the reduced transmission of viruses to other individuals, the establishment of herd protection for those who are not eligible to receive vaccines, health risk protection (i.e., protection against the uncertainty of becoming ill and needing to access health services), and even macroeconomic impacts such as sustained economic activity [53,59,71,72,73,74,75]. It is worth noting that several HTA and NITAGs already consider vaccine coverage rates and herd protection within their value assessment frameworks [40,41,47,48,76,77], allowing for the incremental value introduced by new vaccine platforms in these dimensions to be readily quantified and incorporated through existing methodologies. Nonetheless, estimating the extent to which combination vaccines will affect vaccination rates and projecting their impact in prospective value assessments may prove challenging during the initial rollout of a vaccine. It is important, however, that these value drivers are not entirely disregarded in value assessments, as failing to account for them would result in undervaluation.

## 4. Efficient Manufacturing and Production Processes

New vaccine platforms, such as mRNA, do not require the use of cell cultures and can often develop products faster than traditional vaccine platforms [78]. Producing a traditional vaccine against an infectious disease can take anywhere from 6 months for an influenza vaccine up to 3 years for pentavalent and hexavalent vaccines [79,80,81]. Because only a component of the genetic material of the pathogen is needed to develop an mRNA vaccine, the production process does not include the time-consuming preparation step of growing whole living organisms and expanding cell cultures or the need to inactivate or attenuate the pathogen (which are required steps for developing whole cell-based vaccines). Instead, the genetic material from the viruses can be synthesized via an in vitro reaction, resulting in a more scalable platform with enhanced production efficiency [78,82]. For example, the more efficient mRNA vaccine manufacturing and production processes allowed for the <90 day timeline from the FDA’s strain recommendation for updated COVID-19 vaccines on 15 June 2023 to the vaccines being available to the public by 14 September 2023 [23,24].

With fewer opportunities for supply chain disruptions and improved thermostability profiles (although cold-chain management remains essential), manufacturers and distributors are able to deliver a greater number of vaccines in a timely manner to urban and rural regions alike [83,84,85]. Furthermore, much like with the impact of combining multiple vaccines into a single injection, the ability to develop mRNA vaccines on a larger scale and faster timeline is likely to increase vaccine uptake and coverage [13,54,86]. In fact, vaccine coverage rates are already a key input in many health economic evaluations (e.g., CEAs that incorporate dynamic disease transmission rates and budget impact models) and value frameworks utilized by many NITAGs (e.g., the United States, United Kingdom, France, and Germany) [40,41,43,45,48,77]: improved coverage will lead to better clinical effectiveness both as projected in these models and as achieved in the real world [87,88]. 

## 5. Discussion and Conclusions

The proper clinical and economic evaluation of vaccination programs recognizes their broadly administered use and resulting population-wide benefits [1,5,6,11,78,89]. These include reduced disease transmission, reduced fear of contagion, increased health system resilience, and a broad range of non-health economic benefits such as of lower indirect costs, human capital accumulation, and labor market productivity. While value drivers such as vaccine coverage rates, clinical effectiveness, and levels of herd protection are key inputs currently used in models estimating the economic value of vaccine technologies, some or all of these additional aspects are left unaccounted for. Conceptually, many of the benefits of the new scientific advancements associated with mRNA- and nanoparticle-based vaccine platforms could be incorporated within existing value frameworks through updates to those very same inputs. A key challenge for payors and policy makers is, however, accurately calibrating updated inputs to ensure reliable estimates of value and adapting frameworks if necessary to better reflect these unique system-wide aspects.

Researchers will likely need to rely on multiple strategies to collect the necessary information. First, the careful review of existing literature may shed light on the impact of combination vaccine regimens on vaccination uptake, and on the effect of reductions in supply chain disruptions on the population reach of vaccination campaigns. Second, primary research—both qualitative and quantitative—with public health officials, policy makers, manufacturers, physicians, and individuals could be used to estimate the behavioral and logistical responses to more robust vaccine availability, improved accessibility, and the use of combination vaccines. This research can be conducted using existing methods, such as state preference methods and discrete choice experimental study designs [90,91,92]. Third, expert panels could be used to elicit reasonable estimates of vaccine efficacy in the context of later strain matching, for example, using an online-modified Delphi method to circumvent real-world limitations, such as the restricted ability to gather in-person per the traditional Delphi panel method [93]. Finally, in lieu of robust real-world data, scenario and sensitivity analyses could be used to evaluate the sensitivity of value estimates to different modeling assumptions. Even without precise estimates for the effect of each of the dimensions of the value described above, failing to include them would clearly result in underestimates of the value.

The COVID-19 pandemic has been both a wake-up call regarding the potential broad-ranging value of vaccines, as well as a showcase of the significant potential associated with new vaccine platforms. To incentivize the continued development of new vaccines and ensure their widespread availability, it is important that the value frameworks utilized by HTAs and NITAGs properly reflect the full range of health benefits and cost efficiencies that they provide, and that these be considered during coverage and reimbursement discussions.

## Data Availability

No new data were created or analyzed in this study. Data sharing is not applicable to this article.
